# Early discharge and home treatment of patients with acute pulmonary embolism in the tertiary care setting

**DOI:** 10.1007/s11739-023-03415-4

**Published:** 2023-09-05

**Authors:** Stephan Nopp, Julia Bohnert, Thomas Mayr, Daniel Steiner, Helmut Prosch, Irene Lang, Wilhelm Behringer, Karin Janata-Schwatczek, Cihan Ay

**Affiliations:** 1 Clinical Division of Haematology and Haemostaseology, Department of Medicine I, Medical University of Vienna, Waehringer Guertel 18-20, 1090 Vienna, Austria; 2 Department of Biomedical Imaging and Image-Guided Therapy, Medical University of Vienna, Vienna, Austria; 3 Clinical Division of Cardiology, Department of Medicine II, Medical University of Vienna, Vienna, Austria; 4 Department of Emergency Medicine, Medical University of Vienna, Vienna, Austria

**Keywords:** Pulmonary embolism, Emergency department, Home treatment, Risk assessment, Venous thromboembolism

## Abstract

Acute pulmonary embolism (PE) is a potentially life-threatening disease. Current guidelines suggest risk-adapted management. Hospitalization is required for intermediate- and high-risk patients. Early discharge and home treatment are considered safe in the majority of low-risk patients. In this study, we describe characteristics, discharge, and outcome of outpatients diagnosed with acute PE at a tertiary care center. All outpatients undergoing computed tomography pulmonary angiography or ventilation/perfusion lung scan between 01.01.2016 and 31.12.2019 at the University Hospital Vienna, Austria, were screened for a PE diagnosis. Electronic patient charts were used to extract characteristics, clinical course, and outcomes. Within the 4-year period, 709 outpatients (median age: 62 years, 50% women) were diagnosed with PE. Thirty-three (5%) patients were classified as high-risk, 159 (22%) as intermediate-high, 332 (47%) as intermediate-low, and 185 (26%) as low-risk PE according to the European Society of Cardiology risk stratification. In total, 156 (22%) patients (47% with low-risk and 20% with intermediate-low-risk PE) were discharged as outpatients and received home treatment. Rates for home treatment increased 2.4-fold during the study period. Thirty-day mortality in the entire population was 4.9%. All low-risk patients and all but one patient with home treatment survived the first 30 days. Home treatment significantly increased over time and seems to be safe in routine clinical practice. Notably, one in five intermediate-low-risk patients was discharged immediately, suggesting that a subpopulation of intermediate-low-risk patients may also be eligible for home treatment.

## Introduction

Acute pulmonary embolism (PE) is a potentially life-threatening disease with a 30-day mortality rate of approximately 10% [1–3]. However, a significant proportion of PE patients are at low risk for adverse outcomes. In those, hospitalization and monitoring are not required and these patients may be eligible for ambulatory/home treatment of acute PE. The introduction of direct oral anticoagulants (DOAC) a decade ago and their ease of use further facilitated immediate or early discharge. Nevertheless, less than 10% of PE patients were selected for home treatment until recently [4–7].

As of 2014, guidelines started raising awareness for the possibility of home treatment in carefully selected patients with low risks of adverse outcomes (e.g., low risk for death, recurrent PE, major bleeding) [8–10]. Several randomized controlled trials and prospective management cohort studies followed and provided evidence on the feasibility and safety of using decision tools such as the Pulmonary Embolism Severity Index (PESI), simplified PESI (sPESI), or Hestia rule to decide on the discharge management [11–14]. Based on these studies, the European Society of Cardiology (ESC) PE guidelines of 2019 now recommend risk-adapted management for discharging patients with acute PE. To distinguish between low, intermediate, and high risk for early adverse outcomes, clinical findings, imaging, and biochemical markers need to be taken into account [15]. Patients categorized as having high or intermediate-risk PE should be hospitalized. Low-risk PE patients may be selected for early discharge or home treatment if (i) no serious comorbidity or aggravating condition is present and (ii) proper outpatient care can be provided. The PESI- or sPESI-based approach or the Hestia rule can be used as decision tools. Evidence on the necessity of right ventricular (RV) assessment is unclear, but the guidelines state that it may be wise to exclude RV dysfunction and right heart thrombi if an immediate or early discharge is pursued [15].

Prompted by those changes in guidelines, we sought to investigate the management practice at our university hospital. We aimed to provide a comprehensive descriptive overview of all outpatients diagnosed with acute PE at our tertiary care center and report patient characteristics, disposition management, and mortality outcomes. In more detail, we focused on the clinical practice of early discharge and home treatment in patients at low- or intermediate-low-risk for adverse outcomes.

## Methods

### Study design and population

We conducted a retrospective single-center cohort study of patients diagnosed with PE between January 1st, 2016 and December 31st, 2019 at the Vienna General Hospital, a tertiary care center in Vienna, Austria. Imaging reports of all patients undergoing computed tomography pulmonary angiography (CTPA) or ventilation-perfusion (V/P) lung scan were individually reviewed and screened for PE diagnosis. Patients who were already hospitalized at PE diagnosis due to other causes were excluded. The study was approved by the local ethics committee (EK-Nr: 2330/2020) and conducted in accordance with the declaration of Helsinki. Written informed consent was waived due to the retrospective nature of the study. 

### Data and sources

Patient demographics, characteristics, comorbidities, and data on the PE event including symptoms, vital parameters, and risk factors were individually extracted from electronic patient records including notes from outpatient clinics and wards, imaging reports, and discharge letters. Laboratory parameters at diagnosis (i.e., hs-Troponin T, NT-proBNP, and creatinine) were extracted electronically using a research documentation and analysis software. Survival status and date of death were retrieved from the Austrian National Death Registry and the cause of death in patients who died within the first 30 days was assessed using electronic patient charts and death certificates. The study period ending in 2019 was chosen as the COVID-19 pandemic led to changes in patient flow and management and was therefore not representative of routine clinical practice [16].

### Definitions and outcomes

The severity of the PE event was categorized using the ESC risk assessment strategy [15]. RV dysfunction was defined as present, if either echocardiography and/or CTPA showed RV dysfunction or impairment. Intermediate-risk patients with RV dysfunction and no measurement of hs-Troponin T but an NT-proBNP level above 600 pg/ml were categorized as intermediate-high-risk PE patients. Note that patients with an sPESI of 0 but signs of RV dysfunction were categorized as intermediate-risk patients according to the ESC guidelines. The presence of a risk factor for PE was assessed according to the criteria suggested by the International Society on Thrombosis and Haemostasis (ISTH) [17]. The sPESI score and the Hestia criteria were used as defined in the original studies [18, 19]. Home treatment was defined as immediate discharge from the emergency department or the outpatient clinic. Early discharge was defined as discharge before or on the third day after PE diagnosis. Thirty-day mortality rates are reported as the main outcome.

Other than official PE guidelines, there was no specific internal protocol or strategy for discharge management and follow-up after immediate discharge during the study period. Catheter-based therapies, such as catheter-directed thrombolysis, for selected high- or intermediate-high-risk patients were introduced at our hospital beginning in 2018.

### Statistical analysis

Descriptive statistics on patient demographics, characteristics, disposition, and outcomes are presented using median (25th to 75th percentile, i.e. interquartile range (IQR)) for continuous variables and frequencies (percentages) for categorical variables. Missing values are shown in brackets in Table 1. As intended, inferential statistical analysis was not conducted. All analyses were performed using R (Version 4.1.0; R Core Team, 2021).

**Table 1:** Characteristics, management, and outcomes of outpatients diagnosed with acute pulmonary embolism at a tertiary care center between 2016 and 2019

	Total cohort (n = 709)	ESC risk stratification			
		Low-risk (n = 185)	Intermediate-low-risk (n = 332)	Intermediate-high-risk (n = 159)	High-risk (n = 33)
**Demographics**					
Age, years	62 (49–74)	50 (37–63)	65 (51–75)	68 (55–77)	70 (60–77)
Female sex	356 (50.2%)	87 (47.0%)	164 (49.4%)	85 (53.5%)	20 (60.6%)
**Comorbidities**					
Arterial hypertension	320 (45.1%)	52 (28.1%)	153 (46.1%)	93 (58.5%)	22 (66.7%)
Diabetes	77 (10.9%)	9 (4.9%)	40 (12.1%)	22 (13.8%)	6 (18.2%)
Atrial fibrillation	55 (7.8%)	2 (10.8%)	23 (6.9%)	26 (16.4%)	4 (12.1%)
Chronic heart failure	31 (4.4%)	0 (0%)	11 (3.3%)	19 (12.0%)	1 (3.0%)
Chronic lung disease	92 (13.0%)	0 (0%)	64 (19.3%)	23 (14.5%)	5 (15.2%)
Chronic kidney disease	73 (10.3%)	6 (3.2%)	50 (15.1%)	14 (8.8%)	3 (9.1%)
Chronic liver disease	19 (2.7%)	0 (0%)	13 (3.9%)	5 (3.1%)	1 (3.0%)
History of cancer or active cancer	187 (26.4%)	0 (0%)	141 (42.5%)	40 (25.2%)	6 (18.2%)
Active cancer	137 (19.3%)	0 (0%)	103 (31.0%)	28 (17.6%)	6 (18.2%)
History of VTE	193 (27.2%)	60 (32.4%)	88 (26.5%)	41 (25.8%)	4 (12.1%)
PE	113 (15.9%)	31 (16.8%)	53 (60.2%)	25 (15.7%)	4 (12.1%)
DVT	74 (10.4%)	25 (13.5%)	33 (9.9%)	16 (10.1%)	0 (0%)
Other	6 (0.8%)	4 (2.2%)	2 (0.6%)	0 (0%)	0 (0%)
**PE characteristics**					
Site					
Unilateral	273 (38.5%)	86 (46.5%)	155 (46.7%)	28 (17.6%)	4 (12.1%)
Bilateral	436 (61.5%)	99 (53.5%)	177 (53.3%)	131 (82.4%)	29 (87.9%)
Location					
Subsegmental	85 (12.0%)	38 (20.5%)	35 (10.5%)	11 (6.9%)	1 (3.0%)
Segmental	256 (36.1%)	79 (42.7%)	145 (43.7%)	26 (16.4%)	6 (18.2%)
Lobar	136 (19.2%)	40 (21.6%)	67 (20.2%)	19 (11.9%)	10 (30.3%)
Main	40 (5.6%)	8 (4.3%)	17 (5.1%)	14 (8.8%)	1 (3.0%)
Central	192 (27.1%)	20 (10.8%)	68 (20.5%)	89 (56.0%)	15 (45.5%)
Infarct pneumonia	93 (13.1%)	27 (14.6%)	42 (12.7%)	19 (11.9%)	5 (15.2%)
**PE severity**					
Risk stratification					
Low-risk	185 (26.1%)	/	/	/	/
Intermediate-low	332 (46.8%)	/	/	/	/
Intermediate-high	159 (22.4%)	/	/	/	/
High-risk	33 (4.7%)	/	/	/	/
Right ventricular dysfunction	237 (33.4%)	0 (0%)	48 (14.5%)	159 (100%)	30 (90.1%)^b^
sPESI > 0 points	433 (61.1%)	0 (0%)	269 (81.0%)	131 (82.4%)	33 (100%)
PE diagnosed during anticoagulation	48 (6.7%)	14 (7.6%)	24 (7.3%)	9 (5.7%)	1 (3.0%)
**Risk factors for PE**^a^					
Major transient risk factor	70 (9.9%)	25 (13.5%)	28 (8.4%)	14 (8.8%)	3 (9.1%)
Minor transient	100 (14.1%)	36 (19.5%)	33 (9.9%)	28 (17.6%)	3 (9.1%)
Persistent	171 (24.1%)	17 (9.2%)	116 (34.9%)	32 (20.1%)	6 (18.2%)
None	368 (51.9%)	107 (57.8%)	155 (46.7%)	85 (53.5%)	21 (63.6%)
**Laboratory parameters**					
hs-Troponin T [169], ng/l	18.0 (7.0–51.3)	5.0 (4–8)	16 (8–35)	51 (30 -92)	112 (66–247)
NT-proBNP [147], pg/ml	302 (88–1875)	80 (31–160)	241 (82–1053)	2404 (827–5507)	1858 (388–6890)
**Management and outcomes**					
Management					
Home treatment	156 (22.0%)	87 (47.0%)	68 (20.5%)	1 (0.6%)	0 (0%)
Hospitalized and early discharge (≤ 3 days)	88 (12.4%)	30 (16.2%)	48 (14.5%)	10 (6.3%)	0 (0%)
Hospitalized (> 3 days)	465 (65.6%)	68 (36.8%)	216 (65.1%)	148 (93.1%)	33 (100%)
Number of days hospitalized for admitted patients	7 (5–12)	5 (3–7)	7 (5–11)	8 (6–12)	24 (14–28)^c^
Death at 30 days	35 (4.9%)	0 (0%)	11 (3.3%)	9 (5.7%)	15 (45.5%)

Values are presented as median (interquartile range) or number (percentage). Number in square brackets indicate missing values

^a^If a transient and a persistent risk factor was present, patients were counted in both categories

^b^In one high-risk patient, PE was not the cause of hemodynamic instability. In two patients, RV was classified as absent at initial presentation

^c^In the high-risk group, the number of days hospitalized for admitted patients is only shown for survivors and ranges to 166 days

## Results

### Study population

A total of 7026 patients underwent CTPA or V/P lung scan at our tertiary care center between 01/2016 and 12/2019. Of those 969 patients were diagnosed with acute PE. Two hundred-sixty (26.8%) patients were already inpatient at diagnosis, i.e., hospitalized due to other causes before PE diagnosis. The remaining 709 (73.2%) were outpatients and included into the study.

PE patients were in median 62 (IQR 49–74) years old, 356 (50.2%) were women, and the majority of patients had at least one comorbid condition. In brief, 31 (4.4%) had a history of heart failure, 92 (13.0%) had a history of chronic lung disease, 73 (10.3%) had chronic kidney disease, 50 (7.1%) had a history of cancer, 137 (19.3%) had active cancer, and 193 (27.2%) had a history of venous thromboembolism (VTE) (Table 1).

The diagnosis of acute PE was confirmed based on the results of CTPA in 636 (89.7%) patients and ventilation-perfusion lung scan in 73 (10.3%) patients. The PE event was bilateral in 436 (61.5%) patients and in 192 (27.1%) the most proximal thrombus in the pulmonary vasculature was located centrally. Right ventricular dysfunction, assessed via echocardiography and/or CTPA, was present in 237 (33.4%) patients. About 13% (93 patients) had accompanying infarct pneumonia. PE occurred in 70 (9.9%) patients in the presence of a major transient risk factor. A persistent risk factor such as active cancer, active autoimmune disease or known thrombophilia was present in 171 (24.1%) patients. No provoking risk factor was identified in 368 (51.9%) patients.

### Risk-stratification, discharge management, and outcome of outpatients diagnosed with acute pulmonary embolism

**Fig. 1:**
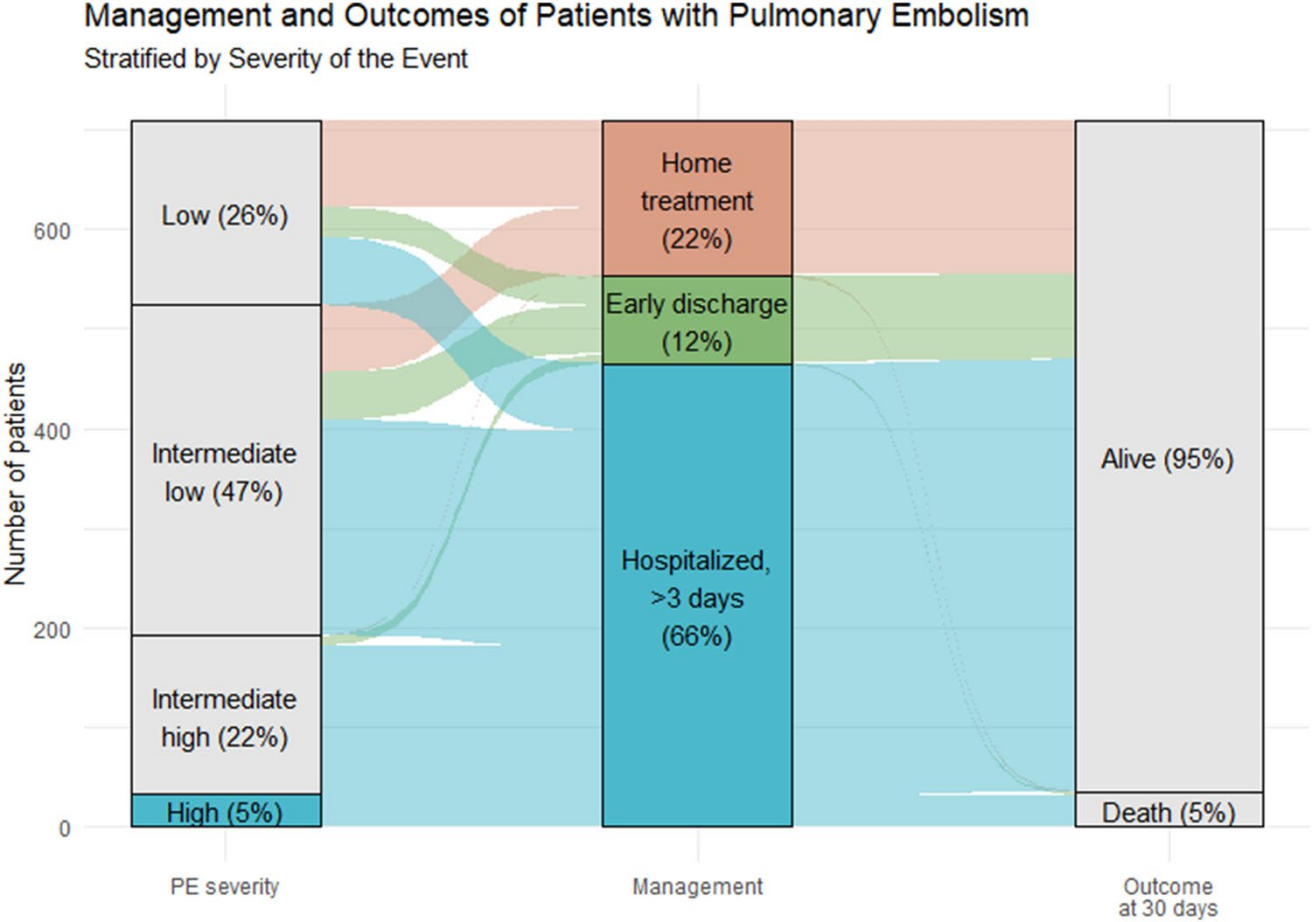
The discharge management and mortality outcomes of patients diagnosed with acute pulmonary embolism between 2016 and 2019 are stratified by the severity of the event according to the European Society of Cardiology Pulmonary Embolism guidelines 2019. High-risk patients are depicted in the same color as patients hospitalized longer than 3 days, as all high-risk patients were managed according to this category

In Fig. 1, management and outcome of all PE patients stratified by the ESC severity assessment are shown. Of all patients, 185 (26.1%) were categorized as having low-risk PE, 332 (46.8%) as intermediate-low-risk, 159 (22.4%) as intermediate-high-risk and 33 (4.7%) as high-risk PE. Of the total study population, 156 (22.0%) were selected for home treatment, 88 (12.4%) were discharged early, and 465 (46.9%) were admitted to hospital longer than 3 days. The median length of hospitalization for admitted patients was 7 days (IQR 5–12). Sixteen (10.3%) patients who received home treatment had some extent of RV dysfunction at diagnosis. Of all patients, 35 (4.9%) died within 30 days following PE diagnosis. 30-day mortality rates for the low, intermediate-low, intermediate-high and high-risk population were 0%, 3.3%, 5.7%, and 45.5%, respectively. 30-day mortality rates for those with home treatment and early discharge were 1.3% and 2.3%, respectively.

**Fig. 2:**
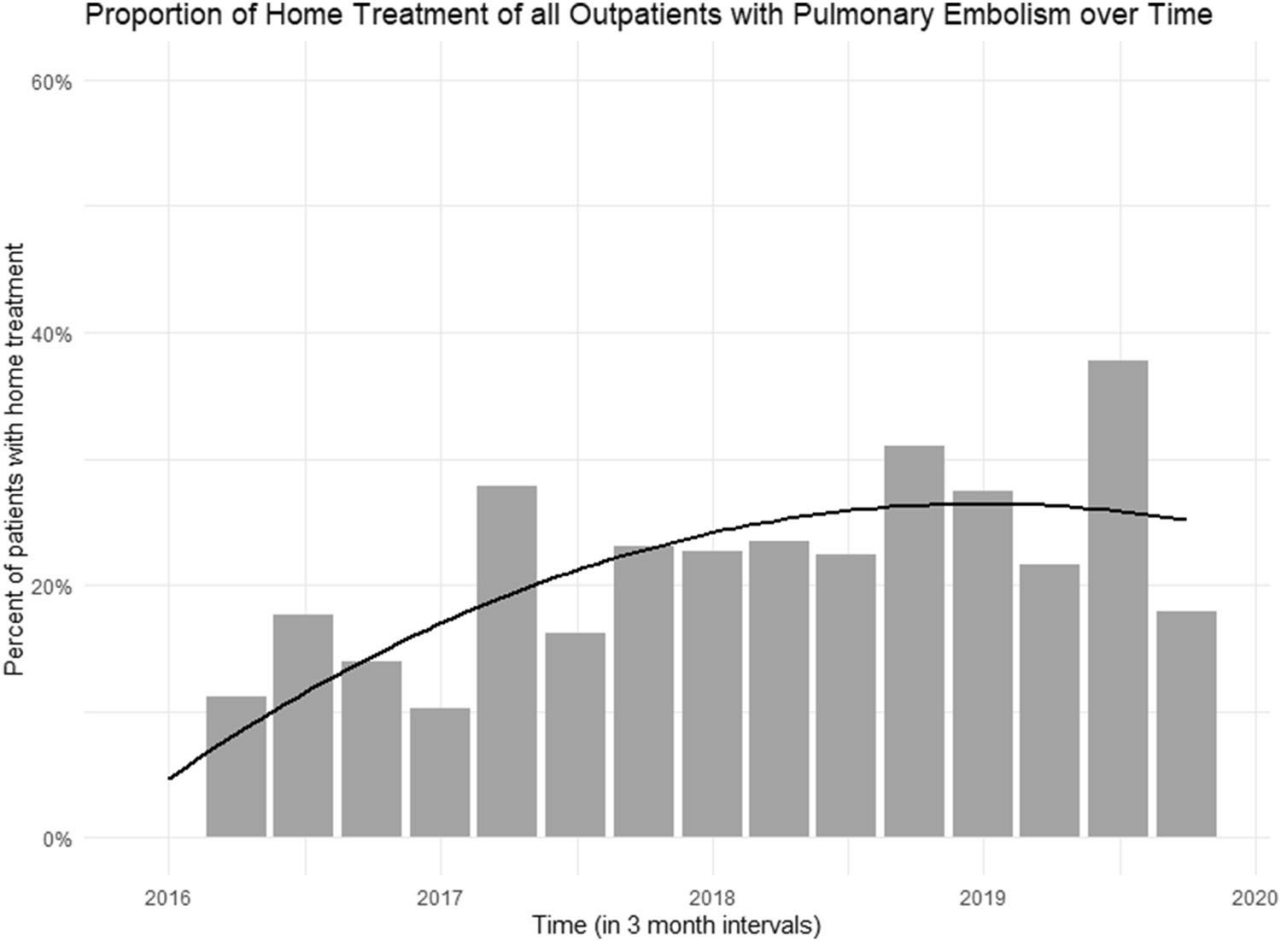
The proportion of home treated outpatients diagnosed with acute pulmonary embolism increased from 11.1% in 2016 to 26.1% in 2019

Figure 2 displays the proportion of patients selected for home treatment over time. During the study period, home treatment increased by 2.4-fold from 11.1% in 2016 to 26.1% in 2019. In the following section, results of outpatient management with regard to home treatment and early discharge are presented in more detail.

### Discharge management and outcome of outpatients with low or intermediate-low-risk pulmonary embolism

In a subgroup analysis, we focused on discharge management of patients with low or intermediate-low-risk PE. In total, 517 (72.9%) patients had low or intermediate-low-risk PE, of which 248 (48.0%) had an sPESI of 0 and 196 (37.9%) had a negative Hestia rule.

Patients with low or intermediate-low-risk PE are characterized, separated based on the discharge management, in more detail in Table 2. Of patients with low-risk PE, 87 (47.0%) patients were selected for home treatment while 98 (53.0%) were admitted to hospital. Per definition, all had an sPESI score of 0. In hospitalized patients, 31 (31.6%) had no Hestia criterion present; in those with home treatment, 78 (89.7%) did not meet any of the Hestia criteria. Central PE and infarct pneumonia were more common in the hospitalized than in the immediately discharged group (17.3% vs. 3.4% and 18.4% vs. 10.3%). In the hospitalized low-risk population, 30 (30.6%) were discharged within 3 days. No patient with low-risk PE died during 30-day follow-up.

**Table 2:** Characteristics and outcome of patients with low or intermediate-low-risk acute pulmonary embolism (PE) by discharge management strategy

	Low-risk PE (n = 185)		Intermediate-low-risk PE (n = 332)	
	Home treatment (n = 87)	Hospitalized (n = 98)	Home treatment (n = 68)	Hospitalized (n = 264)
**Clinical decision support tools**				
sPESI, 0 points	87 (100%)	98 (100%)	15 (22.1%)	48 (18.2%)
No criteria of the Hestia rule present	78 (89.7%)	31 (31.6%)	57 (83.8%)	30 (11.4%)
**Patient characteristics**				
Age	48 (38–61)	51 (36–64)	65 (48–77)	65 (51–74)
Female sex	38 (43.7%)	49 (50.0%)	31 (45.6%)	133 (50.4%)
Arterial hypertension	24 (27.6%)	28 (28.6%)	25 (36.8%)	128 (48.5%)
Diabetes	5 (5.7%)	4 (4.1%)	6 (8.8%)	34 (12.9%)
Atrial fibrillation	0 (0%)	2 (2.0%)	5 (7.4%)	18 (6.8%)
Chronic heart failure	0 (0%)	0 (0%)	0 (0%)	11 (4.2%)
Chronic lung disease	0 (0%)	0 (0%)	11 (16.2%)	53 (20.1%)
Chronic kidney disease	2 (2.3%)	4 (4.1%)	4 (5.9%)	46 (17.4%)
Chronic liver disease	0 (0%)	0 (0%)	1 (1.5%)	12 (4.5%)
History of cancer or active cancer	0 (0%)	0 (0%)	22 (32.4%)	119 (45.1%)
Active cancer	0 (0%)	0 (0%)	11 (16.2%)	92 (34.8%)
History of VTE	21 (24.1%)	39 (39.8%)	22 (32.4%)	66 (25.0%)
**PE characteristics and severity**				
Central PE	3 (3.4%)	17 (17.3%)	6 (8.8%)	62 (23.5%)
Bilateral PE	36 (41.4%)	63 (64.3%)	32 (47.1%)	145 (54.9%)
RV dysfunction	0 (0%)	0 (0%)	15 (22.1%)^b^	33 (12.5%)
Infarct pneumonia	9 (10.3%)	18 (18.4%)	8 (11.8%)	34 (12.9%)
**Risk factors for PE**				
Major transient risk factor	10 (11.5%)	15 (15.3%)	3 (4.4%)	25 (9.5%)
Minor transient	14 (16.1%)	22 (22.4%)	7 (10.3%)	26 (9.8%)
Persistent	5 (5.7%)	12 (12.2%)	15 (22.1%)	101 (38.3%)
None	58 (66.7%)	49 (50.0%)	43 (63.2%)	112 (42.4%)
**Laboratory parameters**				
hs-Troponin T > 14 ng/l	0 (0%)	0 (0%)	11 (25.0%)^a^	113 (58.5%)^a^
NT-proBNP > 600 pg/ml	0 (0%)	0 (0%)	4 (10.3%)^a^	72 (34.1%)^a^
**Management and outcomes**				
Early discharge (≤ 3 days)	/	30 (30.6%)	/	48 (18.2%)
30-day mortality	0 (0%)	0 (0%)	1 (1.5%)	10 (3.8%)

^a^Percentage was calculated from the number of patients with available measurements. hs-Troponin T was not measured in 24 patients with home treatment and 71 hospitalized patients. NT-proBNP was not measured in 29 patients with home treatment and 53 hospitalized patients

^b^Among the 15 intermediate-low-risk PE patients discharged with right ventricular (RV) dysfunction, three showed RV dysfunction as confirmed through both echocardiography and computed tomographic pulmonary angiography (CTPA). Five patients displayed RV dysfunction when assessed by CTPA, but not by echocardiography. Additionally, in seven patients, RV dysfunction was identified solely through CTPA, as echocardiography was not conducted in these individuals

Of patients with intermediate-low-risk PE, 68 (20.5%) received home treatment and 264 (79.5%) were admitted to hospital. The proportion of patients with an sPESI score of 0 were fairly similar between both groups (22.1% vs. 18.2%). In contrast, the proportion of patients who did not meet criteria of the Hestia rule was much higher in the home treatment population (83.8% vs. 11.4%). Central PE was more common in the hospitalized than in the home treatment cohort (23.5% vs. 8.8%). In intermediate-low-risk PE patients with home treatment, 11 (16.2%) had a history of cancer, an additional 11 (16.2%) had active cancer, and 15 (22.1%) showed sign of RV dysfunction in echocardiography and/or CTPA. Hospitalized patients with intermediate-low-risk PE were discharged early in 18.2% (48/264) of cases. Of patients with intermediate-low-risk PE, 11 (3.3%) died during 30-day follow-up. Of those, one received home treatment, 7 died after discharge and 3 died during hospitalization. The deceased patient with home treatment was hospitalized 6 days after PE diagnosis for planned treatment initiation for severe pulmonary hypertension and further received an unsuccessful percutaneous treatment for peripheral artery occlusion during the hospital stay. Twenty-six days after PE diagnosis, the patient died due to spontaneous massive retroperitoneal bleeding in hospital that could not be stopped despite surgical intervention.

Notably, also one patient (0.6%) with intermediate-high-risk PE had home treatment and 10 (6.3%) patients were discharged early within the first three days. The intermediate-high-risk patient with home treatment had end-stage cancer and immediate discharge was the patient’s choice against the recommendation of the treating physician.

## Discussion

In this study involving 709 acute PE patients, we investigated clinical practice of early discharge and home treatment at our tertiary care center and report the highest home treatment rates among unselected PE patients. Home treatment of patients diagnosed with acute PE increased from 11 to 26% between 2016 and 2019. Half of low-risk PE patients were selected for home treatment but also one in five patients with intermediate-low-risk were discharged immediately following PE diagnosis. Overall, home treatment and early discharge were safe. All low-risk patients survived the first 30-day period.

Acute PE is potentially life-threatening, and the decision to discharge a patient is challenging given medical, ethical, and legal concerns. However, prolonged hospitalization is a major burden for patients and health care systems. Outpatient/ambulatory care is associated with cost savings [20–22] and probably most PE patients even benefit from immediate or early discharge [23], given the reduced risk of hospital-acquired infections and iatrogenic complications [21, 24]. Earlier discharge and return to normal physical and professional activity may also limit functional decline, especially in the elderly [25]. Thus, ambulatory care of normotensive (non-high-risk) patients should be preferred whenever possible [23]. Head-to-head comparisons from a randomized-controlled trial of inpatient versus outpatient care for acute low-risk PE patients proved non-inferiority regarding safety and efficacy of home treatment [22, 26]. Evidence for the safety of home treatment is further supported by prospective cohort and management studies [11–14, 27, 28].

In our retrospective cohort study, we could confirm the high level of safety of home treatment in patients with low-risk PE outside of clinical trials. Thirty-day mortality rate was at 0% in both, hospitalized and immediately discharged low-risk patients. However, still half of low-risk patients were hospitalized in our study. Although home treatment-rates in our study were more than twice as high compared to recently published studies [4–7, 28] (including also a pragmatic effectiveness trial conducted during the same study period [14]), the 2019 ESC guideline recommendations for the management of PE suggest that a significant proportion of hospitalized low-risk patients in our study could have been selected for home treatment [15]. Taken together, home treatment in low-risk patients is still underused in clinical practice, but the time trends in our study suggest further increases in home treatment-rates, especially with the strengthening of guidelines recommendations for home treatment in 2019 and recent management trials.

The ESC guideline recommends admitting intermediate-risk patients. However, in our study, one in five intermediate-low-risk patients were immediately discharged after PE diagnosis, including cancer patients, elderly, and patients with RV dysfunction or elevated cardiac biomarkers. All but one of home treated patients survived the first 30 days. The deceased patient died 26 days after PE diagnosis, unrelated to the decision for discharge, and after being hospitalized for 20 days for a planned stay. Our findings suggest that home treatment and early discharge are safe also in a carefully selected population with intermediate-low-risk PE. This data also adds up to the recently published HOME-PE study, a clinical trial that assessed the optimal discharge support tool [29]. In this study, 739 patients were safely selected for home treatment, of whom also a considerable proportion were elderly (5% were above 80 years of age), patients with active cancer or a history of cancer (12%), patients with chronic heart failure or chronic lung disease (6%), and patients with RV dysfunction (12%). It seems that the current guideline approach to recommend hospitalization is very conservative and the recommendation for home treatment might be extended also to a specific population of intermediate-low-risk PE patients. Such considerations are further supported by a systematic review on the role of RV dysfunction, in which the authors conclude that outpatient/ambulatory treatment of hemodynamically stable PE patients seems to be associated with a lower rate of adverse event than inpatient treatment, regardless of their initial risk stratification [23].

Deciding whom to admit and whom to discharge is an ongoing debate, and several validated decision support tools are available. The guidelines, driven by the findings of the HoT-PE study [11], recommend the usage of the PESI or sPESI score, which are also incorporated in the severity risk stratification scheme, in conjunction with assessing the feasibility of early discharge. Alternatively, the Hestia criteria alone may be used. The recently published HOME-PE trial now provides evidence on the head-to-head comparison of the sPESI vs. the Hestia strategy [29]. While more patients were proposed for home treatment in the sPESI strategy than with the Hestia rule (similar to our study), the actual number of patients treated at home was about 37% in both strategies. Importantly, outcomes did not differ between both strategies. Thus, sPESI and Hestia rules may be equally used. In our study, the vast majority of intermediate-low-risk patients selected for immediate discharge had either an sPESI of 0 points or a negative Hestia rule, but not both. Possibly a combined approach of both currently recommended strategies could identify a subgroup of intermediate-low-risk patients also eligible for home treatment.

The importance of RV dysfunction assessed with echocardiography, CTPA, or laboratory markers for the decision to initiate home treatment in low-risk patients is still a matter of discussion. In earlier trials, patients with RV dysfunction were specifically excluded from being eligible for discharge [11]. In the very recent HOME-PE trial, patients with RV dysfunction were also eligible for discharge. Notably, none of the 90 patients with RV dysfunction in this trial experienced adverse outcomes during follow-up. Similarly, 15 patients with RV dysfunction were discharged safely in our study. Thus, we would argue that although signs of RV dysfunction are associated with increased mortality [30, 31], those patients should not be dogmatically excluded from home treatment. Presumably, whether RV dysfunction was preexisting should also be taken into account. Importantly, the low concordance between CTPA and echocardiography in identifying RV dysfunction [32], coupled with differences in prognostic values of specific imaging signs (e.g., subhepatic contrast reflux into inferior vena cava was a strong predictor of mortality while others were not [33]) suggests an oversimplification in treating RV dysfunction as a binary variable. A more nuanced understanding and refinement of RV dysfunction could enhance the selection process for PE patients suitable for home treatment. Another important subpopulation are patients with cancer, who are excluded from home treatment when using the sPESI strategy. However, in the HOME-PE trial and in our study, patients with cancer could be safely selected for discharge. More prospective data on this large subpopulation of PE patients with cancer regarding outpatient management is needed to refine home treatment recommendations.

Our study is limited by several issues related to its retrospective nature. Retrospective assessment of variables such as “medical or social reason for admission to hospital”, a Hestia criterion, are prone to bias as the assessor might be influenced by the factual decision of admitting or discharging a patient. No general approach is recommended to evaluate the safety of discharge management in acute PE. We have reported and interpreted 30-day mortality outcomes. More data on the presence of deep vein thrombosis, bleeding complications, recurrent VTE events and factors such as patient preference or usage of decision support systems would likely add valuable information on the evaluation of safety but could not be reliably assessed across all patients in our study. Advantages of the retrospective approach are that, in contrast to clinical trials, we could include all consecutive patients diagnosed with PE. Together with the moderate-to-large number of patients, this study can provide a comprehensive overview of clinical practice in PE patients treated at a tertiary care center in a European country.

## Conclusion

Home treatment of acute PE substantially increased during the study period but might still be underused in clinical practice. No safety concern regarding home treatment occurred in our study. Notably, one in five intermediate-low-risk patients was discharged immediately, suggesting that immediate and early discharge of acute PE patients might be extended to a carefully selected intermediate-low-risk population. Future efforts should be directed on how to identify intermediate-low-risk patients eligible for discharge.
